# Carbapenemases in Enterobacteriaceae: Detection and Antimicrobial Therapy

**DOI:** 10.3389/fmicb.2019.01823

**Published:** 2019-08-20

**Authors:** Xiaoyan Cui, Haifang Zhang, Hong Du

**Affiliations:** Department of Clinical Laboratory, The Second Affiliated Hospital of Soochow University, Suzhou, China

**Keywords:** carbapenem-resistant Enterobacteriaceae, CRE, prevalence, rapid detection, treatment

## Abstract

Carbapenem-resistant Enterobacteriaceae (CRE) have spread rapidly around the world in the past few years, posing great challenges to human health. The plasmid-mediated horizontal transmission of carbapenem-resistance genes is the main cause of the surge in the prevalence of CRE. Therefore, the timely and accurate detection of CRE, especially carbapenemase-producing Enterobacteriaceae, is very important for the clinical prevention and treatment of these infections. A variety of methods for the rapid detection of CRE phenotypes and genotypes have been developed for use in clinical microbiology laboratories. To overcome the lack of efficient antibiotics, CRE infections are often treated with combination therapies. Moreover, novel drugs and emerging strategies appeared successively and in various stages of development. In this article, we summarized the global distribution of various carbapenemases. And we focused on summarizing and comparing the advantages and limitations of the detection methods and the therapeutic strategies of CRE primarily.

## Introduction

Carbapenem antibiotics are generally considered the most effective antibacterial agents for the treatment of multidrug-resistant bacterial infections. However, with the widespread use of carbapenem antibiotics, the prevalence of carbapenem-resistant Enterobacteriaceae (CRE) has increased rapidly, and has become a serious threat to public health. The production of carbapenemases is the major mechanism underlying carbapenem resistance in CRE throughout the world. Carbapenemases are a kind of β-lactamase that can hydrolyze carbapenem antibiotics. According to the Ambler classification method, carbapenemases can be divided into classes A, B, and D. Class A and class D carbapenemases are serine β-lactamases, and class B carbapenemases are metallo-β-lactamases (MBLs) (Ambler, 1980). There is a large overlap between CRE and carbapenemase-producing Enterobacteriaceae (CPE), but the difference is that they were named according to the carbapenem-resistant phenotype and the resistance mechanism (carbapenemase production), respectively. The correct distinction of CRE and CPE and the rapid detection of CPE are important in the treatment and management of clinical infections. This article summarizes the epidemiology of CRE, the detection of CPE, and the status of clinical treatments.

## Epidemiological Analysis of CRE

The widespread distribution of CRE is mainly attributable to their production of carbapenemases and the plasmid-mediated horizontal transmission of the encoding genes. The prevalence of CRE and the carbapenemase species involved are highly dependent upon the geographic region.

In 2001, the United States first reported a *Klebsiella pneumoniae* (KPN) strain carrying a plasmid-mediated carbapenemase gene encoding a protein later designated *K. pneumoniae* carbapenemase (KPC) (Yigit et al., 2001). From then on, *bla*_KPC_ have spread widely in the United States and South America. And the outbreaks of KPC-producing Enterobacteriaceae are reported in majority of European regions successively (Munoz-Price et al., 2013; Patel and Bonomo, 2013). In China, the first KPC-producing CRE strain was identified in 2007 (Wei et al., 2007), and since then, *bla*_KPC–__2_ has become the most widely spread carbapenemase gene (Zhang et al., 2017). KPN was the main clinically isolated CRE producing KPC. Among the KPC-producing KPN, multilocus sequence typing (MLST) of most strains is clonal complex 258 (CC258), which indicated that CC258 obtained a KPC-encoding gene in the early epidemic of CRE and spread rapidly (Bowers et al., 2015). The predominant sequence type (ST) in China is ST11, and ST258 is predominant in the United States while ST340, ST437, and ST512 predominate in other countries (Chen et al., 2014). Therefore, clonal transmission is considered the main mechanism by which KPC-producing KPN is disseminated.

In 2009, *bla*_NDM_-associated carbapenem-resistant KPN was first reported in India (Yong et al., 2009). Since then, *bla*_NDM_ has been detected in most species of Enterobacteriaceae (Tsang et al., 2012; Berrazeg et al., 2014). NDM-type β-lactamase mainly spread in Asia like India, Pakistan, Bangladesh, especially in China (Dortet et al., 2014). In recent years, NDM has become the second commonest carbapenemase found among CRE in China (Zhang Y. et al., 2018), and *bla*_NDM_ is more prevalent in *Escherichia coli* (Zhang et al., 2017). Due to the horizontal transfer of epidemic broad-host-range plasmids (Pitout et al., 2015), a high diversity of *bla*_NDM_-associated *E. coli* has been detected, among which ST131, ST167, and ST410 are the dominant types (Zhang et al., 2017). Besides, *bla*_*IMP*_ have spread throughout Japan since the IMP-1 was first discovered in Okazaki (Ito et al., 1995). At present, IMP-producing Enterobacteriaceae were found in Japan and Taiwan, China with the highest frequency (Nordmann et al., 2011). In other countries, the outbreaks or reports of *bla*_*IMP*_ are sporadic (Bush and Jacoby, 2010; Nordmann et al., 2011; Patel and Bonomo, 2013). As for VIM, Greece is the epicenter of VIM-producing Enterobacteriaceae (Walsh et al., 2005). Certainly, there are significant outbreaks in other parts of Europe such as the United Kingdom, Belgium, Spain, Italy, Hungary, and some Asian regions such as Taiwan, China, and South Korea. Moreover, the sporadic outbreaks of VIM-producing Enterobacteriaceae are globally reported (Walsh et al., 2005; Vatopoulos, 2008; Nordmann et al., 2011; Glasner et al., 2013).

**FIGURE 1:**
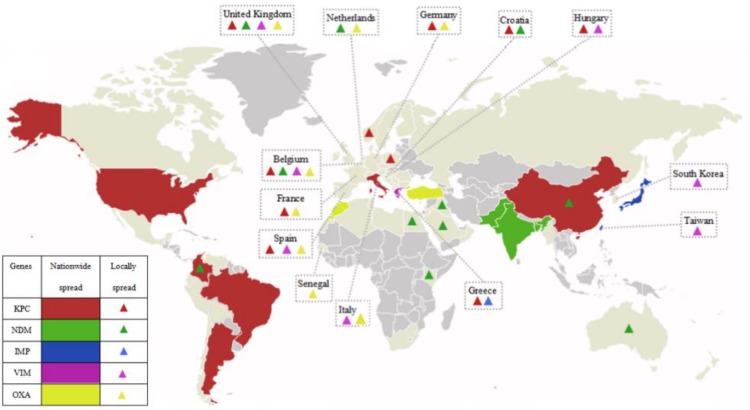
The global distribution of various carbapenemases in CPE. Carbapenemases have emerged in majority regions all over the world. KPCs are the most common carbapenemases and mainly prevalent in China, the Unite States, Italy, and the majority regions of South America; NDMs are mainly prevalent in China, Pakistan, India, and Bangladesh, and widely spread around the world; IMPs are mainly prevalent in Japan and Taiwan, China; VIMs are mainly prevalent in Greece; OXA mainly refers to OXA-48, and is mainly prevalent in Turkey, Morocco, and European countries (France, Germany, Netherlands, Italy, the United Kingdom, and so on); and various carbapenemases locally spread in Europe.

The class D β-lactamases, which function by splitting oxacillin, are designated oxacillinases (OXA). In 1985, the first OXA-encoding gene was found in an *Acinetobacter baumannii* isolate from the United Kingdom and designated *bla*_OXA–__23_ (Donald et al., 2000). Since then, a number of OXA family members have gradually been detected in the Enterobacteriaceae, including OXA-23-like, OXA-48-like, OXA-40-like, OXA-51-like, and OXA-58-like (Evans and Amyes, 2014). The commonest class D β-lactamases is OXA-48, which was first identified in a KPN isolate from Turkey in 2001 (Poirel et al., 2004). OXA-48 includes classical OXA-48 and its variants, OXA-181 and OXA-23 (Pitout et al., 2015). CRE producing OXA-48 are mainly concentrated in European countries (France, Germany, Netherlands, Italy, the United Kingdom, and so on), Middle East (Turkey), and Mediterranean countries, including North Africa (mainly Morocco, Tunisia, Egypt, and Libya) (Stewart et al., 2018). Figure 1 has shown the global distribution of CRE that produce various carbapenemases.

In the past few years, cases of multiple carbapenemases in the same Enterobacteriaceae isolate have been reported. For example, *bla*_NDM–__1_ and *bla*_*IMP–*__4_ coexisted in KPN (Chen et al., 2015), *Enterobacter cloacae* or *Citrobacter freundii* carried both *bla*_KPC_ and *bla*_NDM_ (Feng et al., 2015; Du et al., 2016a; Yang et al., 2018). Besides, there was a *Klebsiella oxytoca* isolate coexpressing three carbapenemases, KPC-2, NDM-1, and IMP-4, which was identified in 2017, and the plasmids containing these three resistance genes have emerged in most other members of the family Enterobacteriaceae, including *E. coli*, *E. cloacae*, and *Klebsiella* species (Wang et al., 2017).

## Rapid Detection of Carbapenemases

Initial susceptibility testing like broth microdilution techniques, the Kirby–Bauer disk diffusion method and automatic analysis systems were standardized and simple. But using the screening breakpoints recommended by the CLSI or EUCAST guidelines will miss the inefficient carbapenemases like KPC variants and OXA-48 (Fattouh et al., 2016; Gagetti et al., 2016). Automated systems may cause discrepancies in the detection of all types of carbapenemase producers (Woodford et al., 2010). Therefore, phenotypic assays and molecular-based techniques are the two main methods currently used to detect carbapenemases.

### Phenotypic Detection Assays

The modified Hodge test (MHT) is a common phenotypic method for the detection of CPE. It is based on whether the growth of the indicator strain is enhanced at the junction of the inhibition zone and the growth line produced by the indicator strain and the test strain, respectively, and estimates whether the test strain has an inactivation effect on antibacterial drugs (Girlich et al., 2012). The method has high sensitivity and specificity in detecting KPC-producing CRE but poor sensitivity in detecting class B β-lactamases (<50%). However, this limitation can be overcome by the addition of Triton X-100, which was proposed and called the Triton Hodge test. This method increased the sensitivity of the detection of NDM-producing clinical isolates to >90% and improved its performance in detecting other carbapenemases at the same time (Pasteran et al., 2016). But the false-positive and false-negative results will affect clinical judgment (Carvalhaes et al., 2010).

Nordmann et al. (2012) subsequently developed a colorimetric assay, the Carba NP test, which is faster and has lower false-positive rate than MHT. In this test, the change in the pH of the reaction system caused by the carbapenemase hydrolysis of imipenem is monitored as the concomitant change in the color of phenol red, which is judged subjectively by the operator in the laboratory. Moreover, this method could preliminarily identify carbapenemases types based on tazobactam and EDTA (Dortet et al., 2012). And then Pires et al. (2013) replaced phenol red with bromothymol blue as the pH indicator when they developed the Blue-Carba test, which improved the assay sensitivity from 93.3 to 100% (Novais et al., 2015). Bogaerts et al. (2016) proposed an electrochemical method derived from the traditional assay, and designated it the Bogaerts–Yunus–Glupczynski (BYG) Carba test. This test reduces the time required from 2.5 h to about 30 min, and resulting from the real-time curve results, this test offers a real-time objective measurement of carbapenemase-producing isolates (Bogaerts et al., 2016). Various commercialized products are also available, such as Rapidec Carba NP (bioMérieux), Rosco Rapid Carb Screen, and the Rapid Carb Blue Kit. A study suggested that most manual and commercial rapid colorimetric assays are insufficiently sensitive for the detection of OXA-48-type producers (Tamma et al., 2017). In 2018, another study demonstrated that the MBT STAR-Carba kit (Bruker Daltonics), which is based on bicarbonate, displays higher sensitivity in the detection of OXA, but still cannot avoid undetected errors (Rapp et al., 2018).

The carbapenem inactivation method (CIM) is another effective phenotypic test. This method determines the carbapenemase activity of the tested bacteria by measuring the diameter of the inhibition zone of *E. coli* ATCC 25922 after the carbapenem disk is inactivated by the test bacterium. The results are highly consistent with the presence of carbapenemase genes, including those encoding KPC, NDM, VIM, IMP, OXA-48, and OXA-23, detected with polymerase chain reaction (PCR) (100% agreement for Enterobacteriaceae) (van der Zwaluw et al., 2015). The modified CIM (mCIM) became the CLSI-recommended method in 2017. A study indicated that both the sensitivity and specificity of mCIM were 100% (Kuchibiro et al., 2018). Because of its simplicity, clear criteria, cost-effectiveness, and availability in any laboratory, the mCIM has become a useful tool in microbiology laboratories.

Many tests that rely on directly monitoring the hydrolysis of β-lactamases to detect CPE have been reported, including a spectrophotometric method (Bernabeu et al., 2012), which is regarded as a reliable detection assay. But extracting the carbapenemases from the bacterial cells is time-consuming, and there were various factors reducing the veracity of the results. To overcome these limitations, Takeuchi et al. (2018) developed a dual-wavelength measurement which could measure the hydrolytic activity of carbapenemases using bacterial cells directly. On the one hand, this method is time saving (about 40 min for preparation and incubation, but the time of detecting OXA should be prolonged appropriately). On the other hand, this method showed higher sensitivity and specificity than carbaNP at the same incubation time, and obtained consistent results upon mCIM. However, the requirement for a specific instrument (spectrophotometer) and the small sample size limit its clinical application (Takeuchi et al., 2018).

In 2011, Hrabák et al. (2011) proposed that matrix-assisted laser desorption ionization–time of flight mass spectrometry (MALDI–TOF MS) could be used to screen CPE by detecting the by-products of the hydrolyzed carbapenem. Since then, other groups have developed various MALDI–TOF-based methods to improve the sensitivity of the procedure, reduce the detection time, and facilitate the interpretation of the results (Johansson et al., 2014; Knox et al., 2014; Sauget et al., 2014; Lasserre et al., 2015; Papagiannitsis et al., 2015). For example, aiming at the low sensitivity mainly resulting from the false-negative results obtained with OXA-48-type producers, Papagiannitsis et al. (2015) added NH_4_HCO_3_ to the reaction buffer, which improved its sensitivity from 76 to 98%. To save time, Lasserre et al. (2015) developed a MALDI–TOF-based method that directly detects resistant Enterobacteriaceae from primary culture plates in <30 min and ensures high sensitivity and specificity. In 2018, a survey demonstrated that a MALDI–TOF-MS-based ertapenem hydrolysis assay rapidly and accurately detected the carbapenemase activity of Enterobacteriaceae strains in positive blood cultures (Yu et al., 2018a). Although the costs of measurement using MALDI–TOF MS are low, the equipment remains expensive, which limits the wide application of this method in clinic (Lasserre et al., 2015).

As well as all these methods, carbapenemase-inhibitor-based disc tests have been shown to detect carbapenemases (Tsakris et al., 2010). For example, combining boronic acid with an ertapenem or meropenem disk has been applied in detecting production of KPC (Doi et al., 2008). Adding ethylenediaminetetraacetic acid to a carbapenem disk makes it a useful compound in detecting MBLs (Franklin et al., 2006). Ote et al. developed an immunochromatographic assay to directly detect OXA-48-like carbapenemase using a monoclonal antibody, and the results were obtained in a very short time (Glupczynski et al., 2016). A bioluminescence-based carbapenem susceptibility detection assay was reported in 2018 that allows carbapenemase-producing CRE and non-carbapenemase-producing CRE to be distinguished with a sensitivity of 99% and a specificity of 98% (Van Almsick et al., 2018).

### Molecular-Based Detection Methods

Tests based on molecular techniques are considered the gold standards for the identification of carbapenemase genes (Nordmann et al., 2011), the advantages and limitations have been summarized in Table 1. PCR is the most commonly used traditional molecular genotyping method. However, the traditional PCR method for identifying a single gene is time-consuming. Therefore, multiple PCR that was time-saving with high levels of sensitivity and specificity (Ellington et al., 2016) was proposed and developed. From 2006 to 2012, the multiplex real-time PCR systems have been initially established for the rapid detection of most carbapenemases like KPC, OXA-48 (Swayne et al., 2011), VIM, IMP (Mendes et al., 2006), and NDM (Monteiro et al., 2012). Furthermore, various modified methods were proposed to overcome the inaccuracy caused by the diversity of OXA-48-like carbapenemases (Hemarajata et al., 2015), such as a real-time PCR assay based on a high-resolution melt analysis (Hemarajata et al., 2015), and a multiplex PCR assay using peptide–nucleic acid probes, which could identify resistance genes in a mixture of Enterobacteriaceae isolates with highly efficient (Jeong et al., 2015).

**TABLE 1:** The advantages and limitations of common detection methods.

**Detection methods**	**Advantages**	**Limitations**
**Phenotypic detection assays**		
Modified Hodge test (MHT)	1. Detecting KPC 2. Simple and inexpensive	1. False-positive and false-negative 2. Insufficient for MBLs 3. Time consuming
Colorimetric assay	1. Detecting KPC and most MBLs 2. Type carbapenemases 3. Simple and inexpensive	1. Insufficient for OXA-48 2. Specific reagents 3. Various infecting factors
Modified carbapenem inactivation method (mCIM)	1. Detecting all carbapanemeses 2. Clear criteria of judgment 3. Simple and cost-effectiveness	1. Time consuming
Spectrophotometric method	1. High sensitivity and specificity 2. Time saving 3. Simple and inexpensive	1. Specific instrument (spectrophotometer) 2. Various influencing factors 3. No standard equation and cut-off value 4. Small sample size
MALDI–TOF-based methods	1. Detecting KPC and NDM 2. Time saving 3. Easy to perform 4. Low measurement cost	1. Insufficient for OXA-48 2. No clear protocol and standard analysis 3. Expensive equipment
Molecular-based detection methods	1. Gold standards 2. Detecting all carbapanemeses genes 3. Type carbapenemase genes 4. Time saving	1. High technical requirements 2. Insufficient for expression of genes 3. High measurement cost

As well as the methods described above, several other molecular methods are used to detect CPE. For example, Walker et al. (2016) combined nested PCR, real-time PCR, and microfluidics to identify the common carbapenemases genes. A PCR-based method in a cartridge format developed to detect CPE in rectal swabs, which is run on the GeneXpert platform, displayed high sensitivity (96.6%) and specificity (98.6%) within a short time (32–48 min) (Tato et al., 2016). Srisrattakarn et al. (2017) developed a loop-mediated isothermal amplification method with hydroxynaphthol blue dye (LAMP-HNB), which was highly efficient (100% sensitivity and specificity). In 2018, the microfluidic chip technology which allows the rapid detection of pathogens and their resistance genes (Kim et al., 2017) was used to detect carbapenem-resistance genes, with high sensitivity and specificity (both >90.0%), and fully met the requirements for clinical diagnoses (Zhang G. et al., 2018). Verigene Gram-negative blood culture assay, the microarray-based commercialized products, was available to identify the carbapenemases (Ledeboer et al., 2015). But the materials cost is a little bit expensive approximately $60–80 per test (Hill et al., 2014). In addition, whole genome sequencing is the most reliable method for the detection of carbapenemase genes, but the high cost, long turnaround time, and difficult data management limit the routine clinical application of this method (Patel, 2016). Yu et al. (2018b) also developed a novel multiplex PCR amplification reaction to directly and rapidly identify the epidemic CRKP ST258/ST11 strain. The advantages and limitations of common detection methods have been shown in Table 1.

## Treatment of CRE Infections

To the bests of our knowledge, almost all β-lactam antibiotics have limited effects on the treatment of CRE infections, and carbapenemases cannot be inhibited by traditional β-lactamase inhibitors (Zhang et al., 2017). Some restricted drugs, such as polymyxins, tigecycline, and fosfomycin, may be active. A proportion of CRE strains producing KPC and OXA-48 are also sensitive to aminoglycosides (gentamicin and amikacin). However, there are significant deficiencies in the use of monotherapy to treat CRE infections with these antibiotics. Polymyxin has significant nephrotoxicity and neurotoxicity (van Duin et al., 2013), and the optimal dose for treatment is unknown. This antibiotic has also been challenged by the emergence and global spread of mobilized colistin resistance (mcr) determinants. The presence of both *mcr-1* and various *bla*_NDM_ has been reported in Enterobacteriaceae isolates (Du et al., 2016b; Yao et al., 2016; Zheng et al., 2017; Li et al., 2018). The increased mortality risk conferred by tigecycline (Cai et al., 2010; Shen et al., 2015; Ni et al., 2016) has led to warnings by the Food and Drug Administration (FDA, 2013). Furthermore, reports of clinical tigecycline resistance were published soon after its first use in medical practice. The resistance mechanisms that have been reported including mutations in *tet* (Linkevicius et al., 2016; He et al., 2019) and the increased expression of RND efflux pumps (Nicoloff and Andersson, 2013; Fang et al., 2016). Besides, tigecycline tends to inducing resistance during therapy (Spanu et al., 2012; van Duin et al., 2014; Du et al., 2018). The therapeutic effects of aminoglycosides in CRE infections can be affected by rmtB which confers high-level and widespread resistance (Cheng et al., 2016). The efficacy of fosfomycin is limited and resistance to this drug develops rapidly during treatment (Karageorgopoulos et al., 2012). Moreover, fosfomycin-modified genes play the key role in fosfomycin resistance. It is noteworthy that a carbapenem-, colistin-, and tigecycline-resistant *E. coli* strain carrying the *fosA3* was reported in China in 2018 (Wang et al., 2018), which poses a great threat to public health.

For the reasons described above, several methods have been proposed to enhance the efficacies of these antibiotics, including aerosolized antibiotics for treatment with colistin (Valachis et al., 2015) and higher maintenance doses of colistin and tigecycline (Falagas et al., 2014; Trifi et al., 2016). These regimens did improve the therapeutic effects, but convincing evidence is sparse. In this context, combination therapies have been recommended to treat multidrug-resistant CRE infections. Not only the retrospective studies but also the *in vitro* tests and clinical applications have proved that the combination therapies were effective for the treatment of CRE (Cprek and Gallagher, 2015; Oliva et al., 2015; Ku et al., 2017). And the mortality rates associated with combination therapies especially the carbapenem-containing combinations were lower than those associated with monotherapy (Mataseje et al., 2016). By combining previous researches on combination therapies (Entenza and Moreillon, 2009; Tzouvelekis et al., 2012; Falagas et al., 2014; Pontikis et al., 2014; Chinese XDR Consensus Working Group et al., 2016), several regimens were proposed in Table 2. However, the mechanistic basis of the synergy has not yet been established for most commonly used combination therapies (Baym et al., 2016).

**TABLE 2:** The advantages and limitations of the combination therapies.

	**Combination therapies**	**Advantages**	**Limitations**	**Mechanisms of resistance**
Tigecycline-based combinations	1. +aminoglycosides^a^ 2. +carbapenems^b^ 3. +fosfomycin 4. +polymyxin	1. Effective for kinds of CRE (Sader et al., 2015) 2. Lower mortality rates	1. Unclear mechanism 2. Unclear optimal dose 3. Poor pharmacokinetic properties (Giamarellou and Poulakou, 2011) 4. Side effects were evident with increasing dose (Tasina et al., 2011; Ramirez et al., 2013) 5. Inducing resistance	1. Increasing expression of RND efflux pumps 2. Mobile resistance genes, *tet*(A), *tet*(K), *tet*(M), *tet*(X3), and *tet*(X4) (Linkevicius et al., 2016; He et al., 2019)
Polymyxin-based combinations	1. +carbapenems^b^ 2. +tigecycline 3. +fosfomycin			1. Mobile colistin resistance genes
Other combinations	1. fosfomycin + aminoglycosides^a^ 2. aztreonam + aminoglycosides^a^ 3. Tigecycline + polymyxin + carbapenem^b^			1. Fosfomycin-modified genes and modification of MurA for fosfomycin resistance (Solomkin et al., 2014) 2. rmtB for aminoglycosides resistance

*^*a*^Aminoglycosides refer to amikacin and isepamicin. ^*b*^Carbapenems refer to meropenem and imipenem.*

As well as the antibiotic combination treatments, novel β-lactamase inhibitors and antimicrobial therapeutics were developed to treat CRE infections and eliminate colonization. Avibactam (AVI) is a novel β-lactamase inhibitor that inhibits KPC, ESBL, AmpC, and OXA-48 (van Duin and Bonomo, 2016). Ceftazidime–AVI (CAZ–AVI) has been used in clinical treatments in the United States since 2015 and was recommended by CLSI in 2018. These combination is effective not only for strains producing KPC and OXA-48 (Castanheira et al., 2015), but also for hypervirulent KPN carrying *bla*_KPC–__2_ (Yu et al., 2018c). CAZ-AVI combined with ertapenem also successfully treated a patient infected with NDM-producing KPN (Camargo et al., 2015). And clinical reports indicated that CAZ-AVI showed commendable therapeutic effect in treating complicated urinary tract or intra-abdominal infections (Tuon et al., 2018). Comparing with colistin, CAZ-AVI showed better efficacy, lower mortality, and fewer side effects in treating KPC-producing CRE (van Duin et al., 2018). However, CAZ–AVI-resistant isolates have been reported since 2015 (Humphries et al., 2015; Shields et al., 2017). To broaden the antibacterial spectrum, aztreonam–AVI was proposed, and effectively inhibited a variety of class A, B, and D carbapenemases (Vasoo et al., 2015). Another two novel carbapenem-β-lactamase inhibitor combinations, imipenem–relebactam and meropenem–vaborbactam, were developed to treat CPE infections. And the latter has been recommend by FDA^1^. *In vitro* data have indicated that the two combinations are highly active against KPC-producing Enterobacteriaceae but poorly susceptive against MBLs and OXA-type carbapenemases (Lapuebla et al., 2015a, b). And exact efficacy and safety must be defined with further clinical data (Zhanel et al., 2018). Besides, meropenem–nacubactam during clinical development have shown promising *in vitro* activity against KPC and MBL-producing CRE (Barnes et al., 2019; Mushtaq et al., 2019). Moreover, cefepime–zidebactam could inhibit CRE producing carbapenemases of classes A, B, and D (Thomson et al., 2019), other cefepime-β-lactam enhancer such as cefepime–enmetazobactam (AAI101)/WCK-5153, etc. which were in earlier stages of development may represent a novel carbapenem-sparing option (Giacobbe et al., 2018; Moya et al., 2019; Papp-Wallace et al., 2019). Several other new drugs such as plazomicin, eravacycline, and cefiderocol developed to treat CRE infections are in various stages of development (Kohira et al., 2016; Thaden et al., 2017; Rodríguez-Baño et al., 2018), among which plazomicin performed more potent effect and lower side effects than other aminoglycosides (Livermore et al., 2011; Riddle et al., 2012; Walkty et al., 2014; Castanheira et al., 2018) and eravacycline showed favorable clinical response and had well pharmacokinetics, tolerability, and *in vitro* activity (Zhanel et al., 2016; Thaden et al., 2017). The application of cefiderocol needs further clinical data. In 2018, the injection products of plazomicin and eravacycline have been recommend by FDA^2^. However, due to the emergence of resistant isolates (Livermore et al., 2011; Castanheira et al., 2018; He et al., 2019), enough attention should be paid to the development of drug resistance. The advantages, limitations, and mechanisms of resistance of novel antimicrobial therapeutics have been shown in Table 3.

**TABLE 3:** The advantages and limitations of novel antimicrobial therapeutics.

**Antimicrobial therapeutics**	**Advantages**	**Limitations**	**Mechanisms of resistance**
Ceftazidime–avibactam	1. Inhibition of KPC, OXA-48, ESBLs 2. Effective for CR-hvKp 3. Effective for complicated urinary tract and intra-abdominal infections 4. Low mortality risk (Shields et al., 2016)	1. Poor inhibition of MBLs and the other OXA (Livermore et al., 2016) 2. Unclear efficacy on other infections	1. Mutation of Ompk35/Ompk36 and high expression of KPC and SHV (Nelson et al., 2017) 2. Point mutation (Shields et al., 2017)
Aztreonam–avibactam	1. Inhibition of KPC, MBLs, ESBLs, OXA	1. Insufficient phase III clinical trials data	
Imipenem–relebactam	1. Inhibition of KPC 2. Favorable *in vitro* activity (Lob et al., 2017) 3. Well tolerated (Sims et al., 2017) 4. Few adverse evens (Zhanel et al., 2018)	1. Poor inhibition of MBLs and OXA (Lapuebla et al., 2015a) 2. Insufficient phase III clinical trials data (Sims et al., 2017)	1. Low expression of OmpK36 (Hecker et al., 2015)
Meropenem–vaborbactam	1. Inhibition of KPC (Lapuebla et al., 2015b) 2. Well tolerated 3. Few adverse evens (Zhanel et al., 2018)	1. Poor inhibition of MBLs and OXA (Lapuebla et al., 2015b) 2. Insufficient clinical data support	1. Low expression of OmpK35 and OmpK36 (Ritchie and Garavaglia-Wilson, 2014)
Plazomicin	1. Inhibition of KPC and OXA (Castanheira et al., 2018) 2. More potent activity and lower side effects than other aminoglycosides	1. Poor inhibition of MBLs	1. Methylation of 16S rRNA (Livermore et al., 2011) 2. Aminoglycoside modifying enzyme (Castanheira et al., 2018)
Eravacycline	1. Well pharmacokinetics, pharmacodynamics, tolerability, and *in vitro* activity (Lan et al., 2019; McCarthy, 2019) 2. Performance in complicated intra-abdominal infections (Heaney et al., 2019) 3. Non-renal pathway clearance (Lee and Burton, 2019)	1. Suboptimal in complicated urinary tract infections (Lee and Burton, 2019)	1. Upregulation of efflux pumps (Livermore et al., 2011) 2. Mobile resistance genes, *tet*(X3) and *tet*(X4) (He et al., 2019)
Cefiderocol	1. Inhibition of kinds of carbapenemases 2. Well tolerability 3. High microbiological response rates and eradication rates (Zhanel et al., 2019)	1. Unclear optimal dose 2. Insufficient phase III clinical trials data	

As well as novel drugs, various strategies for the management of carbapenem resistance have recently emerged. For example, based on studies of fecal microbiota transplantation (FMT) and enteric pathogens (Wang et al., 2014; Caballero et al., 2015), FMT was hypothetically suggested to be used as a clearance method for CRE colonized patients, but the feasibility requires further study (Wang et al., 2016; Qazi et al., 2017). Based on research into the mechanisms of antibiotic cytotoxicity (Cheng et al., 2014; Citorik et al., 2014; Dwyer et al., 2014), novel synthetic tools developed for the precise removal of genomic islands have been proposed to replace antibiotic treatments (Vercoe et al., 2013). Immunological-based therapies, such as monoclonal antibodies targeting poly-(-1,6)-*N*-acetyl glucosamine (Skurnik et al., 2016) and cationic antimicrobial peptides (Pan et al., 2015), are also under investigation as substitutes for traditional antibiotics (DiGiandomenico and Sellman, 2015). The ability of predatory bacteria to reduce the primary pathogen in mammalian system has been demonstrated, which suggested the application prospect in clinic (Shatzkes et al., 2016). The advantages and limitations of these main novel strategies have been summarized in Table 4.

**TABLE 4:** The advantages and limitations of the novel strategies.

**Strategies**	**Advantages**	**Limitations**
FMT	1. Restore the intestinal microbiota 2. Reduced CRE colonization	1. Unclear transplant conditions 2. Insufficient theoretical support
Novel synthetic tools	Favorable treatment effect	High technical requirements
Immunological-based therapies	1. Specific target 2. Superior survival outcomes 3. Low risk of resistance (Zendo, 2013)	1. Narrow antibacterial spectrum 2. Insufficient clinical data support
Predatory bacteria	1. Effective against biofilms 2. Effective for recalcitrant infections (Dwidar et al., 2012)	1. Unclear effects on host 2. Insufficient clinical data support

## Summary

In recent decades, CRE have spread widely in various medical institutions around the world, and due to the time-consuming detection methods and limited treatment regimens, the mortality rates among patients are high. Therefore, the timely and accurate detection of CRE, especially CPE, is essential for the clinical treatment and prevention of infections. A variety of phenotypic methods and gene-based methods are available for the rapid detection of carbapenemases, and these are expected to be used routinely in clinical microbiology laboratories. At present, novel antibacterial drugs and emerging strategies which have been recommend or during development, with good activity and safety profiles, are expected to be applied to the clinical treatment of these infections in the near future.

## Author Contributions

HD designed the study. XC and HZ performed data analysis and prepared the manuscript. All authors approved the final manuscript.

## Conflict of Interest Statement

The authors declare that the research was conducted in the absence of any commercial or financial relationships that could be construed as a potential conflict of interest.

## Footnotes

1 https://www.fda.gov/drugs/development-resources/meropenem-and-vaborbactam-injection

2 https://www.fda.gov/drugs/development-resources/notice-updates
